# Bipolar spectrum, hypothyroidism, and their association with chronic fatigue/myalgic encephalomyelitis-like syndrome in long COVID: could they be identified as early determinants?

**DOI:** 10.3389/fpsyt.2025.1623288

**Published:** 2025-08-28

**Authors:** Massimo Tusconi, Serdar M. Dursun, Francesco Pegreffi, Cesar Ivan Aviles Gonzalez, Vanessa Barrui, Michele Fornaro, Luz Alba Hurtado Lujan, Lina Patricia Camacho Nunez, Arley Denisse Vega Ochoa, Felice Curcio, Mauro Giovanni Carta, Giulia Cossu

**Affiliations:** ^1^ University Hospital of Cagliari, Cagliari, Italy; ^2^ Neurochemical Research Unit, Department of Psychiatry University of Alberta, Edmonton, AB, Canada; ^3^ Department of Medicine and Surgery, University of Enna “Kore”, Enna, Italy; ^4^ Department of Mental Health and Addiction, ASL Ogliastra, Lanusei, Italy; ^5^ Department of Neuroscience, Reproductive Science and Odontostomatology, Federico II University of Naples, Naples, Italy; ^6^ Faculty of Health Sciences, Universidad Popular del Cesar, Valledupar, Colombia; ^7^ Faculty of Medicine and Surgery, University of Sassari (UNISS), Sassari, Italy; ^8^ Department of Medical Sciences and Public Health, University of Cagliari, Cagliari, Italy

**Keywords:** long COVID, chronic fatigue syndrome, myalgic encephalomyelitis, hypothyroidism, bipolar disorder, psychiatric comorbidities

## Abstract

**Background:**

Long COVID has been increasingly linked to persistent clinical manifestations, including chronic fatigue syndrome/myalgic encephalomyelitis (CFS/ME). However, the relationship between this syndrome and pre-existing conditions such as bipolar spectrum disorders and hypothyroidism is not yet clearly established. These disorders may influence the regulation of biorhythms and immune function, suggesting a possible role in the predisposition to the development of CFS/ME in the context of long-term COVID-19.

**Objectives:**

This study investigates the prevalence of hypothyroidism and bipolar spectrum disorders in patients with CFS/ME associated with long-term COVID-19. It compares it with pre-pandemic population data to determine whether these conditions may be predisposing factors.

**Methods:**

A case–control design was used to select cases from a clinical trial on CFS/ME in long COVID, while controls were extracted from pre-COVID epidemiological databases. Comparative statistical analyses, including chi-square tests and analysis of variance (ANOVA), were performed to assess significant differences in the frequency of these conditions between both groups.

**Results:**

The clinical sample showed significantly higher prevalence rates of hypothyroidism [27.78% vs. 1.14%; odds ratio (OR) = 33.07; 95% confidence interval (CI): 7.10–153.70] and bipolar spectrum disorders (16.67% vs. 0.2%; OR = 138.4; 95% CI: 36.40–526.43) compared to control populations (*p* < 0.0001 for both). Similarly, individuals screening positive for depressive symptoms (PHQ9 > 9) showed markedly increased odds (55.5% vs. 4.16%; OR = 28.75; 95% CI: 6.52–126.73).

**Conclusion:**

The findings suggest that hypothyroidism and bipolar spectrum disorders may act as predisposing factors in the development of CFS/ME in long-term COVID-19. Identifying these clinical antecedents could facilitate early detection and the development of targeted intervention strategies in at-risk populations.

## Introduction

The spread of the COVID-19 pandemic has led physicians to observe numerous clinical cases emerging weeks or even months after acute SARS-CoV-2 infection, with fatigue being the most frequently reported symptom. In many cases, post-viral fatigue syndrome became the predominant clinical feature, sometimes exceeding the diagnostic threshold for myalgic encephalomyelitis/chronic fatigue syndrome (ME/CFS) in terms of symptom intensity and frequency (1). A meta-analysis found that approximately 80% of individuals developed at least one persistent symptom during follow-up months after the infection, with fatigue being the most prevalent at 6 months post-infection. Additionally, 13% to 23% of individuals previously hospitalized for SARS-CoV-2 infection experienced significant chronic fatigue (2). The National Institute for Health and Care Excellence (NICE) guidelines state that a diagnosis of ME/CFS requires the exclusion of “another condition” (1), raising concerns about the appropriateness of diagnosing ME/CFS in long COVID cases. Furthermore, several conditions frequently associated with chronic fatigue in long COVID, such as major depressive disorder, could independently contribute to severe fatigue syndrome (3). The association of long COVID with certain mental health disorders has led some clinicians to hypothesize a primarily “functional” etiopathogenesis for the condition, similar to past controversies surrounding ME/CFS. This perspective contributed to a degree of skepticism and a potentially discriminatory attitude toward the syndrome, as was previously observed with ME/CFS. The reluctance of some clinicians to fully recognize ME/CFS-like syndromes in long COVID may also be influenced by the lack of pharmacological or non-pharmacological treatments with proven efficacy (1, 4, 5). This therapeutic gap has led to dissatisfaction among individuals affected by long COVID, similar to those with ME/CFS, regarding healthcare systems and medical care (6–8). Within this context, setting aside the still unresolved issues concerning the pathophysiology of ME/CFS, it is crucial to clarify the risk factors and clinical markers (particularly pre-existing ones) associated with ME/CFS-like syndromes in long COVID. Increasing evidence suggests that both bipolar spectrum disorders and hypothyroidism may share common pathophysiological pathways with chronic fatigue syndromes, particularly through dysregulation of immune responses, neuroendocrine axes (including the hypothalamic–pituitary–adrenal and thyroid axes), and circadian biorhythms. These disruptions are known contributors to fatigue, mood instability, and poor resilience to physiological and psychological stressors. Moreover, subthreshold affective temperaments—particularly cyclothymic and hyperthymic traits—have been linked to increased vulnerability to a wide spectrum of psychiatric and somatic conditions. As recently reviewed by Favaretto et al. (2024), affective temperaments may constitute a transdiagnostic diathesis that predisposes individuals to stress-related syndromes, including those emerging in the aftermath of viral infections such as SARS-CoV-2. Against this background, the present study hypothesizes that individuals with pre-existing bipolar spectrum disorders or hypothyroidism may be at increased risk of developing CFS/ME-like syndromes following COVID-19 (9). This study aims to analyze specific clinical conditions observed in a sample of individuals recruited for a clinical trial on ME/CFS-like syndrome in long COVID and to assess the prevalence of these conditions in comparison with data obtained from community surveys conducted within the same population from which the clinical sample was drawn.

## Methods

### Case–control design

The case group consisted of a sample of consecutive attendees who went to the Liaison Psychiatry Center of the University Hospital of Cagliari for consultations sent by the clinical departments of the same hospital and enrolled in a controlled clinical trial. The institutional ethics committee approved the study with protocol number NP/2023/496; ClinicalTrials.gov: ID NCT05793736. Recruitment at the University Hospital of Cagliari, Italy, occurred from February to May 2023. Samples from regional or national communities from the databases of epidemiology surveys carried out in the pre-COVID era (8–10) were employed as control groups. Some community studies from which the results or databases were used required assessing the people’s clinical anamnesis (10, 11). Any diagnoses of fatigue syndromes could, therefore, be excluded. Controls (*N* = 18, female = 15, 83.33%) were attendees consecutively admitted at the Psychiatric Liaison Unit (Centro di Psichiatria di Consultazione e Psicosomatica) of the University Hospital of Cagliari, Italy (sent for consultation from another clinical unit of the same hospital). Admission criteria were 18 years or older, previous respiratory SARS-CoV-2 syndrome with a positive PCR test and fever in the last 12 months, and fulfilling the diagnostic criteria for CFS/ME of the Committee on the Diagnostic Criteria for Myalgic Encephalomyelitis/Chronic Fatigue Syndrome (2015) post-course. Exclusion criteria were intellectual disability with levels from moderate to severe and a minimum level of visuomotor coordination. No exclusion by sex was done. Despite the small sample size, the magnitude of the observed effects, particularly for bipolar disorder [odds ratio (OR) = 138.4], was sufficiently large to ensure adequate statistical power. The small sample size is a key limitation of this study, potentially leading to statistical instability and overestimation of effect sizes—particularly in the case of rare conditions such as bipolar spectrum disorders. While the large ORs observed may reflect true associations, they must be interpreted cautiously due to wide confidence intervals (CIs) and the risk of sampling bias. Nonetheless, the robustness of the findings across both chi-square and Fisher’s exact tests strengthens their credibility. Replication in larger, prospective cohorts is needed to confirm these results and enhance their generalizability. A *post-hoc* power analysis based on the observed data yielded a power of approximately 89%, supporting the validity of the detected differences. Nonetheless, the limited number of cases constrains the generalizability of the findings and warrants replication in larger, independent cohorts. Each participant needs to sign a consent form to participate. The study aimed to measure the difference between the frequency of depressive symptoms of those who screened positive for depression on the sample of long COVID CFS/ME-like syndrome and the frequency of the same parameters in the pre-COVID community sample; a control group was drawn from a community database, aimed to assess the prevalence of depressive episodes by screening methods similar to that adopted in the case sample (12). For each clinical case, a matched block of controls of the same age and sex from the database was created. For each clinical case, a matched block of community controls was constructed based on exact age and sex matching from a pre-COVID epidemiological database. Four controls were randomly selected from each block using a random number generator. Once selected, controls were excluded from subsequent selections to avoid duplication. This 1:4 matching strategy was adopted to maximize statistical efficiency while controlling for key demographic confounders. From each block, four controls were randomly extracted. Once a subject was selected, it was automatically excluded from the remaining selections, as far as the difference between the long COVID sample vs. the community pre-COVID sample in the frequency of hypothyroidism, hypothyroidism, major depressive episodes, bipolar disorder, and panic disorder. Psychiatric diagnoses in clinical and community samples were established using the ANTAS-DSM-IV, a structured clinical interview based on DSM-IV criteria, developed and validated for use in Italian populations. While less internationally known than instruments like the Mini International Neuropsychiatric Interview (MINI) or the Structured Clinical Interview for DSM Disorders (SCID), it has been extensively used in epidemiological research in Italy and has demonstrated adequate sensitivity and specificity in community-based mental health studies. Its selection was based on continuity with prior data sources and its proven feasibility in large-scale population screening within the same geographic and cultural context. While not identical to the MINI or SCID, the ANTAS instrument has been used in numerous epidemiological studies and provides comparable diagnostic reliability in community and clinical settings. We used the community studies already published and the help of the databases of the same studies when still in our possession [from the frequency of bipolar disorders and post-traumatic stress disorder (PTSD) in men] that had used the same diagnostic parameters used by the study on the CSF/ME syndrome, namely:

1. Hypothyroidism (10). The diagnosis of hypothyroidism was conducted through the measurement of TSH, FT3, and FT4. Possible cases of subclinical hypothyroidism (i.e., elevated TSH with normal FT3 and FT4) were not considered; only cases of evident hormone deficiency were considered. This criterion was applied to the general population sample (with a reported prevalence of 1.1%) at the time of evaluation and to the clinical sample before initiating any potential replacement therapy.
2. Bipolar disorders (unpublished data in the community sample). Clinicians diagnosed the clinical sample using ANTAS-DSM-IV according to DSM-IV criteria; the data were unpublished and drawn from the study database. Previously, only the prevalence rate of bipolar spectrum disorder was published according to a screening tool (11, 13). Clinicians diagnosed the clinical sample.
3. Major depressive disorders (14). The diagnosis of the community sample was carried out employing ANTAS-DSM-IV according to DSM-IV criteria. Clinicians carried out the diagnosis of the clinical sample.
4. PTSD (15). The diagnosis in the community sample was carried out by clinicians employing ANTAS-DSM-IV according to DSM-IV criteria; the data concerning male frequencies were unpublished and drawn from the study database. Clinicians diagnosed the clinical sample.
5. Panic disorders (16). Clinicians diagnosed the community sample using ANTAS-DSM-IV according to DSM-IV criteria (17).

The frequencies in the clinical sample were standardized concerning the general population samples with a direct method considering sex (M/F) and age (<45/<44). In the only study on hypothyroidism for which the database no longer existed, a comparison was conducted between the raw frequencies; however, it should be considered that, in our sample, unlike in the general samples, there was a precise higher frequency in men and that these were under-represented. It is, therefore, conceivable that the standardized frequencies were even higher.

### Statistical analysis

Differences in numerical data among groups were carried out using one-way analysis of variance (ANOVA), and the differences between the frequencies in normal data (i.e., in case and controls) were measured between chi-square, with Yates correction if required. Because of the limited sample size, we did not perform multivariate regression analysis. This methodological decision was made to avoid model overfitting and unreliable estimates. However, we acknowledge that the absence of multivariate adjustment restricts our ability to assess the independent contribution of each factor and limits causal inference.

## Results

The clinical sample of people with long COVID and CFS/ME-like syndrome consists of 18 people with a mean age of 49.55 ± 11.58 years, of whom 5 were <45 years old [15 (33.33%)], and 14 were women (83.33%). One of the surveys (12) did not need a medical/anamnestic evaluation of the sample; in this case, it is possible that “hidden” in the control group were some individuals with fatigue syndromes. However, these are sufficiently large samples (72 matched from 1,500 total samples); thus, a condition whose lifetime prevalence was estimated to be approximately 1.5% (18) has been adequately diluted. Furthermore, the presence of pollution in the control group with “cases with the condition” would have, in any case, favored the null hypothesis; this aspect, therefore, does not affect the validity of any differences found between cases and controls. **Table 1** shows the frequency of positive cases on the PHQ9 with the different cutoffs of >4 (including mild depressive symptoms) and <9 (including moderate to severe depressive episodes) and the mean score at PHQ9 in the sample of cases and in the control, sample matched (4/1) drawn from the database of the above-cited community sample. The mean scores at PHQ9 were higher in the control sample (11.27 ± 4.73 vs. 4.15 ± 4.20, *p* < 0.0001) as well as the frequency of people with depressive episodes identified with the two most used cutoff, PHQ9 score >9 (55.5% vs. 4.16%; *p* < 0.0001) and PHQ9 score >4 (94.4% vs. 38.9%; *p* < 0.0001). **Table 2** shows the differences in the frequencies of the disorders analyzed between the clinical sample of people with long COVID and CFS/ME-like syndromes and the different samples of general reference populations evaluated before the COVID era. To account for the small sample size and low event frequency, we conducted Fisher’s exact tests for each binary comparison. The results confirmed the robustness of the observed associations for hypothyroidism (OR = 33.08, *p* = 3.13×10^-^_5_), bipolar disorder (OR = 98.66, *p* = 1.37×10^-^_5_), and PTSD (OR = 47.54, *p* = 4.38×10^-^). The association for major depressive disorder approached significance (*p* = 0.062), while no statistically significant association was found for panic disorder (*p* = 0.487). Given the small sample size and the limited number of cases per cell in some comparisons, Fisher’s exact test was performed alongside chi-square analyses. The results remained statistically significant (all *p* < 0.001), supporting the robustness of the associations despite the sample constraints. Exact *p*-values and CIs are now also reported in the main text for greater clarity. Hypothyroidism presents a significantly higher frequency in the clinical sample (27.78% vs. 1.14%, OR = 33.07; 95% CI: 7.1–153.7) as well as PTSD (27.78% vs. 0.8%, OR = 48.8; 95% CI: 16.54–50.03), bipolar disorder (16.67% vs. 0.2%; OR = 138.4; 95% CI: 36.40–526.43), and major depressive disorder (22.22% vs. 8.5%; OR = 10.72; 95% CI: 4.22–27.21). No statistically significant differences were found in the frequency of panic disorders. Note that it was possible to verify that the onset of all bipolar disorders and cases of hypothyroidism was before having contracted the COVID respiratory syndrome and the CFS/ME-like syndrome. In three cases of major depressive disorders, the onset was after the COVID respiratory syndrome and in likely relation to it; in one case of PTSD, the onset was after the COVID respiratory syndrome and related to the dangers experienced during a severe acute phase. The cases of bipolar disorder had been treated for a long time with valpromide, valproate semisodium, lithium carbonate, and olanzapine, respectively. The cases of hypothyroidism were treated with levothyroxine sodium in four patients and with L-tyrosine in one patient. Two cases of hypothyroidism co-occurred with bipolar disorder in two patients and major depressive disorder in three.

**Table 1:** Mean scores and positive cases on the PHQ9 in long COVID patients with CFS/ME-like symptoms and matched community controls, *n* = 18 vs. *n* = 72 (18 × 4).

Mood symptoms	Cases	Controls	Statistics	*p*	OR (95% CI)
PHQ9, Mean ± SD	11.27 ± 4.73	4.15 ± 4.20	ANOVA 1,88, df; *F* = 39.34	<0.0001	
Detection of the number of people with depressive episodes (PHQ > 9) (at least moderate depressive symptoms)	10 (55.5%)	3 (4.16%)	Chi-square Yates correction 1df = 26.754	<0.0001	28.7 (6.5–126.7)
At least mild (PHQ > 4 to 9) (mild to moderate depressive episodes)	17 (94.4%)	28 (38.9%)	Chi-square Yates 1df = 17.778	<0.0001	26.7 (3.3–213.1)

**Table 2:** Prevalence of psychiatric and endocrine comorbidities in long COVID patients with CFS/ME-like symptoms compared to pre-COVID community samples.

Clinical presentation	Cases [standardized] by sex and age	Controls of pre-COVID community samples	Chi-square 1df with Yates correction	OR (95% CI)
Hypothyroidism	5 [5] (27.78%) [27.78%]	3 (1.14%)	33.842, *p* < 0.0001	33.07 (7.1–153.7)
PTSD	3 [5] (16.67%) [27.78%]	26 (0.8%)	116.80, *p* < 0.0001	48.8 (16.54–50.03)
Bipolar disorder	3 [4] (16.67%) [22.22%]	7 (0.2%)	206.15, *p* < 0.0001	138.4 (36.40–526.43
Major depressive disorder	10 [9] (50.0%) [22.22%]	289 (8.5%)	33.54, *p* < 0.0001	10.72 (4.22–27.21)
Panic disorder	2 [1] (5.55%) (22.22%)	123 (3.6)	0.990, *p* = 0.001	1.56 (0.20–11.86)

## Discussion

Affective temperaments—particularly cyclothymic and anxious subtypes—have been proposed as early markers of mood instability and as vulnerability traits for stress-related conditions. These traits, often present in a subclinical form long before illness onset, may alter physiological responses to chronic stress or immune activation. As highlighted by Favaretto et al. (2024) (9), temperamental profiles may play a key transdiagnostic role across psychiatric and somatic syndromes, including those triggered by post-infectious immune dysregulation. Integrating this dimension into the framework of long COVID may enhance our understanding of individual susceptibility to CFS/ME-like outcomes. Our findings suggest a strong association between hypothyroidism, bipolar spectrum disorders, and CFS/ME-like syndromes in the context of long COVID. While the cross-sectional design precludes causal inference, these conditions may act as predisposing factors or markers of individual vulnerability. Although fatigue is a recognized symptom of both hypothyroidism and mood disorders, the severity and multidimensional nature observed in our sample exceeds typical clinical presentations, supporting the hypothesis of a distinct post-infectious syndrome. Notably, all cases of hypothyroidism and bipolar disorder were adequately treated and clinically compensated prior to SARS-CoV-2 infection. Further longitudinal research is warranted to explore shared pathophysiological mechanisms and to clarify whether these pre-existing conditions amplify susceptibility to CFS/ME-like outcomes following COVID-19. It should be noted that our approach aims to explore a potential pathogenic mechanism common to conditions encompassed within the depressive disorders/bipolar spectrum. In a neo-Kraepelinian perspective (19–21), this spectrum can be understood as a transdiagnostic set of mood-related presentations ranging from normality (22, 23) to states of specific risk (24) to fully diagnosed disorders. These conditions share common determinants, familial predispositions, and patterns of biorhythm dysregulation, often accompanied by periods of hyper-energy or hyperactivity (25, 26). This perspective is shared by many researchers, even though it does not fully align with the categorical approach of official psychiatry. It could be very interesting to discover whether certain conditions could be factors favoring syndromes like CSF/ME because, although the etiology of this disorder is unknown, exploring a common pathogenesis may facilitate both understanding of possible etiological mechanisms and help in the development of therapeutic strategies. The clinical sample showed a markedly higher prevalence of bipolar disorder (16.67% vs. 0.2%, OR = 138.4, 95% CI: 36.40–526.43, *p* < 0.0001) and hypothyroidism (27.78% vs. 1.14%, OR = 33.07, 95% CI: 7.10–153.70, *p* < 0.0001) compared to controls. The study demonstrates a close association between hypothyroidism, mood disorders, and CFS/ME-like syndromes in long COVID. Although some mood disorders arose during long COVID, the cases of bipolar disorders and hypothyroidism were already present before the COVID-19 respiratory syndrome, as were the severe chronic fatigue symptoms that reached a level consistent with a ‘CFS/ME-like’ classification. While CFS/ME diagnostic criteria exclude cases in which symptoms are attributable to other conditions, it remains clinically relevant to investigate whether certain pre-existing disorders—such as depression or hypothyroidism—may increase vulnerability to post-infectious fatigue syndromes. Although both can cause fatigue, the intensity and multisystemic nature of symptoms observed in CFS/ME-like presentations are atypical, even in the context of treated mood or thyroid disorders. Exploring shared pathogenic pathways may ultimately support the development of targeted preventive and therapeutic strategies. It should be noted that the cases presented in this survey, both bipolar disorders and hypothyroidism, were adequately treated and, in compensation, predated the acute phase of the COVID-19 infection. The link between hypothyroidism and bipolar disorder is a recognized fact, although this association is considered to be weak (27, 28). Unfortunately, we do not have data from community surveys conducted in our geographic area that allow us to compare the strength of this association concerning the clinical sample examined. However, the frequencies of comorbidity found in the literature are relatively low. In a retrospective analysis of the data of the US Nationwide Inpatient Sample, a comorbidity of 8.1% hypothyroidism was found in people with bipolar disorder and 9.2% in a clinical sample in Kerala, India (29). In our sample, 75% of bipolar disorders and 77.78% of all mood disorders were associated with hypothyroidism. This close and unique association strongly suggests that the two conditions combined may determine the development of long COVID with CFS/ME. One key limitation of this study is the potential for selection bias due to the differing origins of the case and control groups. While the case group was drawn from participants enrolled in a non-randomized clinical trial, the control group was derived from pre-pandemic epidemiological databases. Although the absence of a perfectly matched control group may limit direct comparability, the clinical trial participants were prospectively recruited using inclusion and exclusion criteria closely aligned with those used in the epidemiological studies. This alignment may help reduce the impact of selection bias, although it cannot eliminate it entirely. The small sample size represents a major limitation of the present study and increases the risk of overestimating effect sizes, particularly in the case of rare exposures or outcomes. The very high ORs observed—such as those for bipolar disorder and hypothyroidism—should be interpreted with caution, as they may partially result from low cell counts. Although the findings remain statistically significant, the magnitude of association is likely inflated and requires confirmation in larger, independent cohorts. We have attempted to mitigate this limitation by including exact *p*-values, CIs, and supplementary analysis using Fisher’s exact test. A major limitation of the study lies in the potential selection bias due to the differing origins of the case and control groups. Another limitation of this study is the lack of multivariate regression analysis to adjust for potential confounding variables. Given the small sample size, conducting a meaningful multivariate model would not yield reliable results due to overfitting and insufficient statistical power. Nonetheless, we accounted for key confounders through careful clinical characterization: all cases of bipolar disorder and hypothyroidism predated the onset of COVID-19 infection and the emergence of CFS/ME-like symptoms. As an additional robustness check, we applied Fisher’s exact test to account for small cell sizes. The test confirmed the significance of key associations, particularly for hypothyroidism, bipolar disorder, and PTSD, reinforcing the validity of our findings despite the limited sample. However, the association with major depressive disorder did not reach statistical significance (*p* = 0.062), and no association was observed for panic disorder. The small sample size limited the use of multivariate regression models, as including multiple covariates would likely result in overfitting and unreliable estimates. While we addressed key confounders by direct standardization for age and sex, and through rigorous clinical assessment of temporal onset, the absence of multivariate adjustment constrains the ability to isolate the independent contribution of hypothyroidism and bipolar disorder. This limitation underscores the need for larger, prospective studies incorporating multivariable approaches to better understand causal pathways. In the case of hypothyroidism, standardization by sex and age was not possible due to the lack of access to raw data from one community dataset. Notably, our clinical sample included a relatively high proportion of men, who are generally less affected by hypothyroidism in the general population. As such, this limitation may have led to an underestimation of the true difference in prevalence. A further limitation lies in the retrospective confirmation of the chronology of comorbidities. While all cases of bipolar disorder and hypothyroidism were clinically established prior to SARS-CoV-2 infection, the lack of longitudinal follow-up data precludes definitive temporal mapping in relation to the onset of CFS/ME-like symptoms. Prospective studies are needed to delineate more precisely whether these conditions constitute true predisposing factors or comorbid amplifiers of post-infectious fatigue syndromes. Importantly, all patients were under pharmacological treatment and clinically compensated prior to the infection, which may have reduced the influence of these comorbidities on the development of post-COVID fatigue syndrome. We do not know and cannot even hypothesize if and how the hypothyroidism–bipolar spectrum association could constitute a determinant or an element of vulnerability towards long-term COVID-19 and, more specifically, the CSF/ME syndrome. Some elements could be explored in depth in future research, such as the role of the known vulnerability of people with bipolar disorder to the dysregulation of biorhythms and social and health rhythms. All patients were on stable pharmacological treatment and clinically compensated prior to SARS-CoV-2 infection, which likely minimized the direct influence of these comorbidities on post-COVID fatigue. Whether the hypothyroidism–bipolar spectrum interaction represents a causal determinant or merely a vulnerability marker remains unclear. Future studies should investigate the potential role of biological rhythm dysregulation—a well-recognized feature of bipolar disorder—and its interplay with social and health-related stressors in shaping susceptibility to long COVID and CFS/ME-like syndromes. Notably, all patients were on stable pharmacological treatment and clinically compensated prior to SARS-CoV-2 infection, reducing the likelihood that pre-existing conditions alone explain the post-COVID fatigue syndrome. While a definitive causal link between the hypothyroidism–bipolar spectrum association and CFS/ME-like outcomes cannot be established, this intersection may signal a latent vulnerability. Future research should investigate whether dysregulation of biological, social, and health-related rhythms, commonly observed in bipolar disorder, modulates susceptibility to post-viral syndromes (30, 31), an element that the pandemic has certainly accentuated (8) and which may have modified the immune response in the hyper-stress phases of the acute infection. On the other hand, the two systems of biological rhythms and thyroid response are closely linked, and it is known that hypothyroidism affects the circadian rhythmicity of melatonin synthesis in a sex-dependent manner (32, 33). Moreover, it is the vulnerability in people with hypothyroidism of another component of biological rhythms, such as body temperature adaptation, that has been seen to be a factor influencing long-term COVID-19 (34, 35). Several converging pathophysiological mechanisms may underlie the association between mood disorders, hypothyroidism, and CFS/ME-like syndromes in long COVID. Neuroinflammation, characterized by elevated pro-inflammatory cytokines, has been implicated in both conditions, potentially disrupting neural circuits involved in fatigue and mood regulation. Dysregulation of the hypothalamic–pituitary–adrenal axis and mitochondrial dysfunction, common to both bipolar disorder and hypothyroidism, may further impair stress resilience and energy metabolism. These overlapping pathways suggest a transdiagnostic vulnerability that may be exacerbated by the immune and metabolic burden of SARS-CoV-2 infection. Furthermore, the relationship seems univocal because a good circadian rhythm and a good melatonin level can directly temper hypothyroidism’s effects and protect from this condition’s negative consequences (36–38).

## Conclusions

This study highlights a potential link between bipolar spectrum disorders, hypothyroidism, and CFS/ME-like syndromes in long COVID, suggesting a shared vulnerability profile rather than a direct causal pathway. Clinically, our findings underscore the importance of recognizing pre-existing endocrine and mood conditions as potential amplifiers of post-infectious fatigue. Future longitudinal research should aim to clarify the temporal dynamics of these associations and incorporate markers of affective temperament, neuroendocrine regulation, and mitochondrial function. Early identification of individuals with vulnerable profiles—through temperament screening and targeted endocrine assessment—may offer new avenues for preventive strategies and personalized interventions in the context of long COVID. The presence of bipolar spectrum disorders and hypothyroidism appears to be strongly associated with the subsequent development of CFS/ME-like syndromes in long COVID, although causality cannot be inferred. Future longitudinal studies are required to explore this relationship more definitively. This association should be carefully studied to understand the mechanisms involved in the pathogenesis of this severe syndrome.

## Data Availability

The original contributions presented in the study are included in the article/supplementary material. Further inquiries can be directed to the corresponding author.
